# Child Mortality Estimation: Consistency of Under-Five Mortality Rate Estimates Using Full Birth Histories and Summary Birth Histories

**DOI:** 10.1371/journal.pmed.1001296

**Published:** 2012-08-28

**Authors:** Romesh Silva

**Affiliations:** Department of Demography, University of California, Berkeley, California, United States of America; Umeå Centre for Global Health Research, Umeå University, Sweden

## Abstract

**Background:**

Given the lack of complete vital registration data in most developing countries, for many countries it is not possible to accurately estimate under-five mortality rates from vital registration systems. Heavy reliance is often placed on direct and indirect methods for analyzing data collected from birth histories to estimate under-five mortality rates. Yet few systematic comparisons of these methods have been undertaken. This paper investigates whether analysts should use both direct and indirect estimates from full birth histories, and under what circumstances indirect estimates derived from summary birth histories should be used.

**Methods and Findings:**

Usings Demographic and Health Surveys data from West Africa, East Africa, Latin America, and South/Southeast Asia, I quantify the differences between direct and indirect estimates of under-five mortality rates, analyze data quality issues, note the relative effects of these issues, and test whether these issues explain the observed differences. I find that indirect estimates are generally consistent with direct estimates, after adjustment for fertility change and birth transference, but don't add substantial additional insight beyond direct estimates. However, choice of direct or indirect method was found to be important in terms of both the adjustment for data errors and the assumptions made about fertility.

**Conclusions:**

Although adjusted indirect estimates are generally consistent with adjusted direct estimates, some notable inconsistencies were observed for countries that had experienced either a political or economic crisis or stalled health transition in their recent past. This result suggests that when a population has experienced a smooth mortality decline or only short periods of excess mortality, both adjusted methods perform equally well. However, the observed inconsistencies identified suggest that the indirect method is particularly prone to bias resulting from violations of its strong assumptions about recent mortality and fertility. Hence, indirect estimates of under-five mortality rates from summary birth histories should be used only for populations that have experienced either smooth mortality declines or only short periods of excess mortality in their recent past.

*Please see later in the article for the Editors' Summary.*

## Abstract 2

Romesh Silva assesses and analyzes differences in direct and indirect methods of estimating under-five mortality rates using data collected from full and summary birth histories in Demographic and Health Surveys from West Africa, East Africa, Latin America, and South/Southeast Asia.

## Editors' Summary

**Background:**

In 1990, 12 million children died before they reached their fifth birthday. Faced with this largely avoidable loss of young lives, in 2000, world leaders set a target of reducing under-five mortality (death) to one-third of its 1990 level by 2015 as Millennium Development Goal 4 (MDG 4); this goal, together with seven others, aims to eradicate extreme poverty globally. To track progress towards MDG 4, experts need accurate estimates of the global and country-specific under-five mortality rate (U5MR, the probability of a child dying before age five). The most reliable sources of data for U5MR estimation are vital registration systems—national records of all births and deaths. Unfortunately, developing countries, which are where most childhood deaths occur, rarely have such records, so full or summary birth histories provide the data for U5MR estimation instead. In full birth histories (FBHs), which are collected through household surveys such as those conducted by Demographic and Health Surveys (DHS), women are asked for the date of birth of all their children and the age at death of any children who have died. In summary birth histories (SBHs), which are collected through household surveys and censuses, women are asked how many children they have had and how many are alive at the time of the survey.

**Why Was This Study Done?:**

“Direct” estimates of U5MRs can be obtained from FBHs because FBHs provide detailed information about the date of death and the exposure of children to the risk of dying. By contrast, because SBHs do not contain information on children's exposure to the risk of dying, “indirect” estimates of U5MR are obtained from SBHs using model life tables (mathematical models of the variation of mortality with age). Indirect estimates are often also derived from FBHs, but few systematic comparisons of direct and indirect methods for U5MR estimation have been undertaken. In this study, Romesh Silva investigates whether direct and indirect methods provide consistent U5MR estimates from FBHs and whether there are any circumstances under which indirect methods provide more reliable U5MR estimates than direct methods.

**What Did the Researcher Do and Find?:**

The researcher used DHS data from West Africa, East Africa, Latin America, and South/Southeast Asia to quantify the differences between direct and indirect estimates of U5MR calculated from the same data and analyzed possible reasons for these differences. Estimates obtained using a version of the “Brass” indirect estimation method were uniformly higher than those obtained using direct estimation. Indirect and direct estimates generally agreed, however, after adjustment for changes in fertility—the Brass method assumes that country-specific fertility (the number of children born to a woman during her reproductive life) remains constant—and for birth transference, an important source of data error in FBHs that arises because DHS field staff can lessen their workload by recording births as occurring before a preset cutoff date rather than after that date. Notably, though, for countries that had experienced political or economic crises, periods of excess mortality due to conflicts, or periods during which the health transition had stalled (as countries become more affluent, overall mortality rates decline and noncommunicable diseases replace infectious diseases as the major causes of death), marked differences between indirect and direct estimates of U5MR remained, even after these adjustments.

**What Do These Findings Mean?:**

Because the countries included in this study do not have vital registration systems, these findings provide no information about the validity of either direct or indirect estimation methods for U5MR estimation. They suggest, however, that for countries where there has been a smooth decline in mortality or only short periods of excess mortality, both direct and indirect methods of U5MR estimation work equally well, after adjustment for changes in fertility and for birth transference, and that indirect estimates add little to the insights provided into childhood mortality by direct estimates. Importantly, the inconsistencies observed between the two methods that remain after adjustment suggest that indirect U5MR estimation is more susceptible to bias (systematic errors that arise because of the assumptions used to estimate U5MR) than direct estimation. Thus, indirect estimates of U5MR from SBHs should be used only for populations that have experienced either smooth mortality declines or only short periods of excess mortality in their recent past.

**Additional Information:**

Please access these websites via the online version of this summary at http://dx.doi.org/10.1371/journal.pmed.1001296.

- This paper is part of a collection of papers on Child Mortality Estimation Methods published in *PLOS Medicine*
- The United Nations Childrens Fund (UNICEF) works for children's rights, survival, development, and protection around the world; it provides information on Millennium Development Goal 4, and its Childinfo website provides detailed statistics about child survival and health, including a description of the United Nations Inter-agency Group for Child Mortality Estimation; the 2011 UN IGME report Levels & Trends in Child Mortality is available
- The World Health Organization has information about Millennium Development Goal 4 and provides estimates of child mortality rates (some information in several languages)
- Further information about the Millennium Development Goals is available
- Information is available about infant and child mortality data collected by Demographic and Health Surveys

## Introduction

In 2001, world leaders, recognizing the lack of progress in reducing under-five mortality and the need for more attention and resources to address the proximate determinants of under-five mortality, agreed to prioritize child mortality reduction as part of the United Nations Millennium Development Goals. In particular, the primary target of Millennium Development Goal 4 is for countries to reduce the level of under-five mortality by two-thirds of 1990 level by 2015.

The most reliable data source for estimating under-five mortality rate (the probability of dying between birth and age 5 y, also denoted in the literature as U5MR and _5_ *q*_0_) at the national level is vital registration data, when such data are complete and timely. Yet, the countries where child mortality remains high or child mortality declines are slow or stagnant are those that lack comprehensive vital registration systems [1]. For these countries, analysts rely heavily on two common forms of retrospective birth history data: full and summary birth histories. In full birth histories (FBHs), which are usually collected via a household survey, women are asked to retrospectively report each live birth, the date of the birth, and, if the child has died, its age at death. In summary birth histories (SBHs), which are mostly collected via a household survey or a population census, women are asked to report only summary information: the number of children ever born to them, the number of those children still surviving at the time of the survey, and proxy exposure information (usually the mother's age, duration of her marriage, or time since her first birth). However, retrospective data in the form of both FBHs and SBHs suffer from a number of limitations [2]. Hence, there are a number of challenging methodological issues in quantifying the magnitude and pattern of under-five mortality rates in many low- and middle-income countries. Analysts can derive direct estimates from FBHs, as they provide detailed information about the date of death and the exposure of children to the risk of dying [3]. In contrast, only indirect estimates are usually constructed from the summary information contained in SBHs, whereby such information as the mother's age are used as proxies for exposure to the risk of dying, to derive under-five mortality rate estimates using model life tables [4]–[7]. Of course, such indirect estimates can, and often are, also be derived from FBHs. The purpose of this paper is 2-fold. The first objective is, using available Demographic and Health Surveys (DHS) data, to provide guidance as to whether to use both direct and indirect analyses from FBHs. The second objective is to investigate whether analysts should use indirect estimates from SBHs at all. This analysis is intended to inform discussions about how to evaluate these two estimation methods and make inferences when the resulting estimates from direct and indirect estimation are inconsistent.

The accurate estimation of under-five mortality rates is important for two fundamental reasons. First, the under-five mortality rate is an important indicator of population health [8]. It is widely used by international agencies to monitor development progress on Millennium Development Goals and other initiatives to improve population health and human welfare [9]. Second, the probability of dying before age 5 y, _5_ *q*_0_, is one of the principal input parameters used to develop estimates of life expectancy at birth and other summary indicators of mortality for developing countries without reliable vital registration [10]. Thus, errors and biases in estimating child mortality lead to notable inaccuracies in other summary mortality measures.

The accurate estimation of under-five mortality rates is difficult in developing countries that lack comprehensive vital registration systems. In such cases, researchers have to rely on direct and indirect estimation methods applied to birth histories collected in ad hoc retrospective surveys and population censuses. Such methods are vulnerable to data errors (inherent in retrospective data collection systems) and errors resulting from simplifying modeling assumptions. For example, one notable data error that can affect direct estimates constructed from FBHs is birth transference. This phenomenon involves the retrospective misreporting of the timing of children's birth dates, which, if systematic across sampled birth histories, can lead to bias in estimates of under-five mortality rates. A key simplifying assumption of the Brass method is the assumption of constant fertility in the recent past. Yet fertility change in recent decades has varied substantially across populations, from relatively small changes in West Africa to notable declines in Asia and Latin America; hence, this simplifying assumption may result in systematic bias.

**Figure 1:**
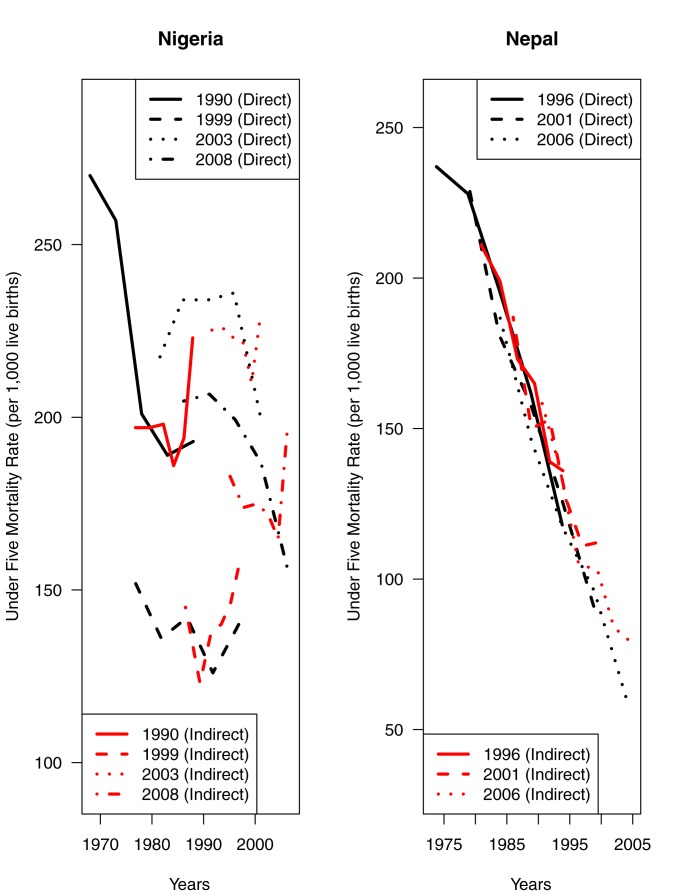
Direct and indirect estimates of under-five mortality rates from available DHS survey data for Nigeria and Nepal.

Further, there is considerable variation in the quantity and quality of DHS survey data for countries in the East Africa, West Africa, Latin America, and South/Southeast Asia regions. To date, there has been only one DHS survey in Sierra Leone, whereas six separate DHS surveys in Senegal have collected both FBHs and SBHs. For illustrative purposes, Figure 1 displays the available DHS survey data on under-five mortality rates for Nepal and Nigeria. The direct and indirect estimates derived from Nepalese DHS data are fairly consistent, whereas the SBHs and FBHs from Nigerian DHS surveys yield highly inconsistent estimates. Such variation in quantity and quality of DHS birth history data poses considerable challenges when attempting to infer temporal patterns and trends in under-five mortality rates in the absence of high-quality vital registration data.

Previous studies on the consistency of direct and indirect estimates of under-five mortality rates have mostly focused on select surveys from the World Fertility Survey conducted in the 1970s and 1980s and on a small number of surveys from early waves of the DHS program. These studies all identified measurable differences between direct and indirect estimates but were not unanimous in their conclusions. Both Preston [11] and Adetunji [12] attributed most of these differences to violations of the assumptions underlying the Brass indirect method. In contrast, the United Nations suggested that in some circumstances indirect methods may still provide useful evidence of levels and trends in under-five mortality rates [13]. Given the increasing importance of and reliance on DHS surveys for estimating under-five mortality in high-mortality countries, this article examines the consistency of FBH and SBH methods of estimating under-five mortality rates.

This paper aims to provide a systematic assessment of when direct and indirect methods result in different estimates, and how best to evaluate such inconsistencies. The paper seeks to provide a sounder empirical basis for the integration of data quality diagnostics into statistical curve fitting of point estimates. Such an integration is needed to ensure that the estimation process better accounts for bias and error in the underlying direct and indirect point estimates.

The primary methodological questions that are addressed in this paper include the following. (1) When FBHs and SBHs are available from the same data source, should analysts use both the direct and indirect estimates in their analysis? If so, which form of the indirect method should be used? (2) Under what (demographic) conditions should direct estimation methods not be used? (3) Under what conditions are indirect methods likely to provide more reliable and valid estimates of under-five mortality rates, or at least sufficiently reliable estimates at substantially lower cost, given that SBH data are less expensive to collect than FBH data?

This comparative study represents a preliminary step towards integrating the early literature of data quality diagnostics and evaluation measures and recent efforts to combine multiple, defective under-five mortality estimates to aid inferential analysis of temporal trends. It assesses direct and indirect estimates by seeking to identify sources of systematic bias and random error in the different estimate types. In particular, potential sources of empirical data error/bias are examined, as well as the errors and bias resulting from violations of underlying model assumptions.

## Methods

### Data Sources

The empirical analysis of this paper is focused on the examination of relative differences between direct and indirect estimates of under-five mortality rates. Direct and indirect estimates are derived from DHS data collected in West Africa, East Africa, South/Southeast Asia, and Latin America. Table 1 lists the data sources used in this analysis.

**Table 1:** Data sources used to examine consistency of direct and indirect under-five mortality rate estimates from a common data source.

Country	DHS Survey(s)
Bangladesh	1993, 1996, 1999, 2004, 2007
Burkina Faso	1992, 1998, 2003
Benin	1996, 2001, 2006
Bolivia	1989, 1993, 1998, 2003, 2008
Brazil	1986, 1991, 1996
Burundi	1987
Congo, Democratic Republic of the Congo	2007
Central African Republic	1994
Congo	2005
Côte d'Ivoire	1994, 1998
Cameroon	1991, 1998, 2004
Colombia	1986, 1990, 1995, 2000, 2004, 2009
Dominican Republic	1986, 1991, 1996, 1999, 2002, 2007
Ecuador	1987
El Salvador	1985
Ethiopia	1985, 1992, 1997
Ghana	1988, 1993, 1998, 2003, 2008
Guinea	1999, 2005
Guatemala	1987, 1995
Guyana	2005, 2009
Honduras	2005
Haiti	1994, 2000, 2005
Indonesia	1992, 2005
India	1987, 1991, 1994, 1997, 2007
Kenya	1988, 1993, 1998, 2003, 2008
Cambodia	2000, 2005, 2010
Liberia	1986, 2006
Sri Lanka	1987
Mali	1987, 1995, 2001, 2006
Malawi	1992, 2000, 2004
Mexico	1987
Nicaragua	1997, 2001
Nigeria	1990, 1999, 2003, 2008
Niger	1992, 1998, 2006
Nepal	1996, 2001, 2006
Peru	1986, 1991, 1996, 2000
Philippines	1993, 1998, 2003, 2008
Pakistan	1990, 2006
Paraguay	1990
Rwanda	1992, 2000, 2005
Sierra Leone	2008
Senegal	1986, 1992, 1997, 2008
Chad	1996, 2004
Togo	1988, 1998
Thailand	1987
Tanzania	1991, 1996, 1999, 2004, 2009
Uganda	1988, 1995, 2000, 2006
Viet Nam	1997, 2002

Source: DHS 1985–2009.

### Methods of Analysis

These differences were evaluated and then successively reanalyzed after diagnostic analysis of and adjustment for various shortcomings of the classical direct and indirect estimation methods. In particular, I successively carried out diagnostic analysis and re-estimation of rates to account for birth omissions and birth transference in FBHs used in direct estimation. I also adjusted indirect estimates by relaxing the implicit constant fertility assumption that underlies the classical Brass indirect method. After each readjustment of the estimates, I successively reviewed the paired differences between the direct and indirect estimates. In order to characterize the observed differences, after controlling for potential data errors in the FBHs and adjusting for violations of assumptions that underlie the Brass method, I drew on categorizations developed by Garenne and Gakusi [14]. In particular, I classified the inconsistencies between revised direct and indirect estimates according to the nature of health and mortality transitions. These classifications distinguish countries by their apparent health transition experience from 1950 to 2000, estimated using pooled direct estimates when several surveys were available for a given country. The classifications organize countries into five main categories: those that experienced (i) a smooth monotonic mortality decline, (ii) minor mortality increases over short periods of time, (iii) major mortality increases due to political and economic crises, (iv) stalled mortality declines for several years, and (v) recent mortality increases due to infectious disease outbreaks. These classifications provide a useful framework to identify the magnitude of observed differences in direct and indirect estimates, relative to the type of health and mortality transition experienced. This helps to identify different inconsistencies observed across different health transitions, particularly when there is deviation from linear mortality decline in the recent past.

I examined the mean relative differences between under-five mortality rate estimates derived using the direct estimation method and the indirect estimation method. Direct estimates were calculated using the synthetic cohort method. Indirect estimates were calculated using two different indirect methods: the classical Trussell version of the Brass indirect method and an adjusted version of the Trussell version that uses cohort-specific parities to relax the constant fertility assumption.

I used paired difference tests to examine whether the indirect estimates were measurably different from corresponding direct estimates. Direct estimates were re-estimated for calendar years corresponding to indirect estimates (associated with SBHs obtained from women aged 25–29, 30–34, and 35–39 y). The calendar years for indirect estimates were derived using the standard method for time referencing of indirect estimates [15].

The relative difference between these two estimation methods, *d_i_*, was defined as follows:
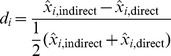
(1)where 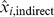 and 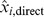 are the estimated rates derived from the indirect method and the direct method, respectively, for the *i-*th year prior to the survey.

I formulated the following hypothesis test to examine whether the mean relative difference between the two estimates was measurably different from zero:
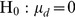
(2)

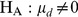
(3)My analysis was focused on differences associated with estimates derived from birth histories of women aged 25–29, 30–34, and 35–39 y. I excluded the SBHs from women aged 15–19 and 20–24 y, since the indirect estimates associated with these age groups are subject to notable selection bias and imprecision. This approach is consistent with the standard methodology used by the United Nations Inter-Agency Group for Child Mortality Estimation [16]. The selection bias for women aged 15–19 and 20–24 y results from the overrepresentation of births to women of low socioeconomic status in these age groups, whereas imprecision results from the notably smaller sample sizes of these birth cohorts. Estimates based on birth histories collected from women older than 39 y were also excluded, because of their vulnerability to notable levels of recall error [17]. I expected any observed difference to, on average, be explained by reliance on model-based age patterns when using the indirect estimation method and by additional data errors and biases to which FBHs may be vulnerable. To accurately compare direct and indirect estimates, direct estimates were centered on the time references associated with the comparable indirect estimates.

## Results

Table 2 displays the results of the paired difference tests using DHS birth histories from West Africa, East Africa, Latin America, and South/Southeast Asia. This analysis is based on DHS data from 132 surveys in 49 countries, as described in Table 1. Surveys from southern Africa were excluded, as such data were likely to be affected by survivorship biases resulting from high HIV prevalence. Data from Australasia were excluded, as they only included Tonga. Data from Europe and Central Asia were excluded, since most of the countries in those regions have attained under-five mortality rate levels below 35 per 1,000. Relative paired differences are reported for mortality estimates associated with SBHs from women aged 25–29, 30–34, and 35–39 y. I observed that the differences were uniformly positive across all regions, and individually statistically significant. This finding is broadly consistent with earlier comparisons that found that, using 15 birth histories collected as part of the World Fertility Survey program, the Brass indirect estimation method resulted in infant mortality rate estimates that were, on average, 20%–35% higher than those derived using the same data source but by employing the direct estimation method [12]. This earlier analysis focused on the following 15 countries: Botswana, Zimbabwe, Namibia, Kenya, Senegal, Burkina Faso, Ghana, Nigeria, Tanzania, Uganda, Zambia, Malawi, and Liberia. These findings suggest that when analysts combine estimates from SBHs and FBHs, they need to factor in such differences to ensure that temporal patterns aren't inferred from error or bias resulting from a particular estimation method.

**Table 2:** Relative paired differences (reported at percentages) between direct and indirect estimates across geographic regions derived using DHS surveys.

Region	Maternal Age Group (Years)	Mean Relative Difference	95% CI
Asia	25–29	9.99	(2.2, 17.8)
	30–34	19.5	(12.5, 26.5)
	35–39	35.0	(26.9, 43.1)
Latin America	25–29	12.7	(7.5, 17.9)
	30–34	23.0	(16.7, 29.4)
	35–39	38.9	(34.3, 43.6)
East Africa	25–29	11.7	(7.6, 15.8)
	30–34	6.9	(1.9, 11.9)
	35–39	10.1	(5.2, 15.1)
West Africa	25–29	6.7	(1.7, 11.6)
	30–34	9.2	(3.3, 15.5)
	35–39	13.7	(8.7, 18.7)
All Regions	25–29	11.5	(8.7, 14.1)
	30–34	17.2	(14.1, 20.3)
	35–39	29.0	(25.9, 32.0)

The paired relative differences for Latin America and Asia tend to be larger than those for West and East Africa. This is likely a result of bias in the Brass indirect method due to violation of the assumption of constant fertility in the recent past, given the, on average, larger fertility declines experienced in Asia and Latin America.

It is therefore important to address the following questions. Are these differences driven by potential data errors in the DHS survey data for West Africa, East Africa, Latin America, and South/Southeast Asia? Or do these differences result from some of the key assumptions of the Brass indirect method being violated for the populations being studied? Or do the differences appear to result from a combination of data errors and underlying methodological assumptions being violated? I investigated whether these observed differences can be explained by apparent data quality issues with the DHS survey data from these regions, and how much of these differences is attributable to the implausibility of the assumptions of the Brass indirect method.

### Diagnostic Analysis of Potential Errors and Biases in Direct and Indirect Estimates

The Brass indirect estimation method can be decomposed into a first component that is affected by model-based errors and a second component that is affected by data errors in the SBHs. This formal decomposition, which is presented in Text S1, is a helpful way of identifying and classifying components of variability in the Brass indirect estimation method.

#### Data errors in birth histories

It is difficult to identify and assess data errors in indirect estimates, in the absence of gold standard data for comparison (e.g., vital registration data). Yet, there are a number of potential data errors that can affect SBHs. Omissions of live births from birth histories are a possible area for indirect estimates to be vulnerable to systematic bias, as the nature of a SBH does not force a mother to recall each live birth individually. However, large survey programs like DHS and Multiple Indicator Cluster Surveys often entail a level of probing and question customization (usually by including questions about children who no longer live with the mother) to minimize such omissions.

In examining SBHs, I focused on evaluations of the recorded female age distribution, sex ratio at birth, and parity distribution. When examining the available data for 49 countries from DHS surveys, I found that data generally conformed to the expected patterns of data quality diagnostics. In general, diagnostic analysis of SBHs did not identify serious flaws in the available data aside from anomalies in sex ratio data in South Asia and some anomalies in female age distributions in conflict-affected countries.

#### Potential data errors and biases in direct estimates

DHS surveys contain two questionnaire modules in which data are collected on antenatal, delivery, and postnatal care of the mother for recent births and on numerous health and nutrition issues for the children born. These modules of the questionnaire must be administered for each birth that occurred after a predefined cutoff date, typically set to January of the fifth complete calendar year prior to a survey (but for some surveys in DHS Phase III set to January of the third calendar year prior to the survey).

For all births that occurred subsequent to the predefined cutoff date, the DHS survey contains approximately 100 additional questions on maternal and child health. Field staff in DHS surveys can considerably lessen their workload by recording births that actually occur after the cutoff date as occurring before that date and, in turn, avoiding the administration of additional survey modules that apply to child births occurring after the cutoff date. Some analysts have documented such birth transference in DHS surveys [18]. Such birth transference can affect mortality rates if it happens in a frequent and systematic fashion and particularly if it affects dead and alive children differently.

Possible displacement of births beyond the reference date in the maternal and child health sections can be discerned by tabulating a birth concentration index, as follows:
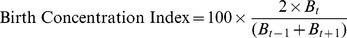
(4)where *B_t_*_−1_, *B_t_*, and *B_t_*_+1_ are the number of births in the *t*−1, *t*, and *t*+1 calendar years.

This index should be close to 100. A value of less than 100 implies fewer births than expected for year *t*.

The birth concentration index is calculated (i) when *t* is the last year for which a child is eligible for the maternal and child health sections and again (ii) when *t* is the year prior to the last year of eligibility for these sections. The last year of eligibility for these sections is defined to be the fifth year preceding the survey for most DHS surveys, and the third year for a few surveys (usually carried out in DHS Phase III).

**Figure 2:**
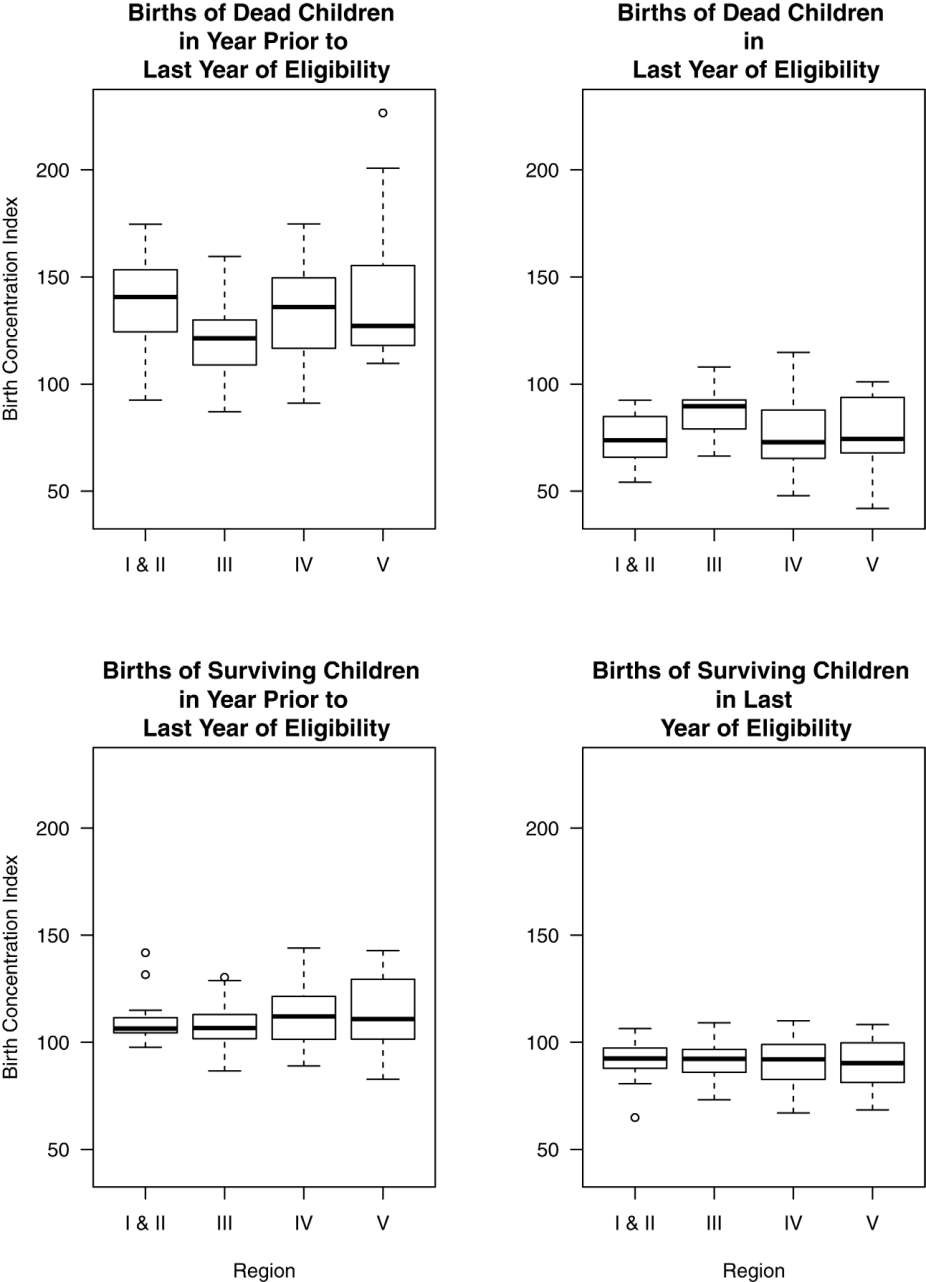
Birth transference for births of surviving and dead children. Birth concentration indexes by DHS phase for births of surviving and dead children in the last year of eligibility for the survey health module and in year prior to the last year of eligibility.

Figure 2 displays a box plot of the displacement ratios for births of surviving and dead children across different phases of the DHS program for the surveys listed in Table 1. I observed that there is a notably higher concentration of births in the year prior to the last year of eligibility for the health sections documented for children who died than those who survived. Also, the extent of birth transference appears to have increased in Phases IV and V of the DHS program, relative to earlier phases of the program.

For estimates pertaining to the 5-y period immediately before the survey, such birth transference is likely to cause a systematic downward bias in under-five mortality rate estimates. For estimates pertaining to the penultimate 5-y period, it is likely to result in a systematic upward bias.

### Adjustment of Direct and Indirect Estimates for Possible Data Errors

I investigated whether the effect of data errors in FBHs and the main assumptions of the Brass indirect method explain the differences between direct and indirect estimates observed in Table 2. I did this by successively examining paired differences after adjusting for data errors in FBHs and then for some of the main assumptions of the Brass indirect method. I adjusted for birth transference by re-estimating the direct estimates for the time period that immediately preceded January of the year prior to the health cutoff for the given birth history. These re-estimated rates capture births (and deaths) that have been transferred across the health module cutoff date. The expected effect of this re-estimation is an increase in the mortality rate for the estimation period immediately preceding the survey and a decrease in the rate for the penultimate estimation period.

I adjusted for potential violations of the constant fertility assumption of the Brass method by recalculating the Brass indirect estimates using cohort-specific parities available in the FBHs of the DHS surveys. By using cohort-specific parities instead of the aggregated period parities, I adjusted for changes in fertility over time instead of assuming constant fertility in the recent past, as is the case in the Trussell version of the Brass method.

**Figure 3:**
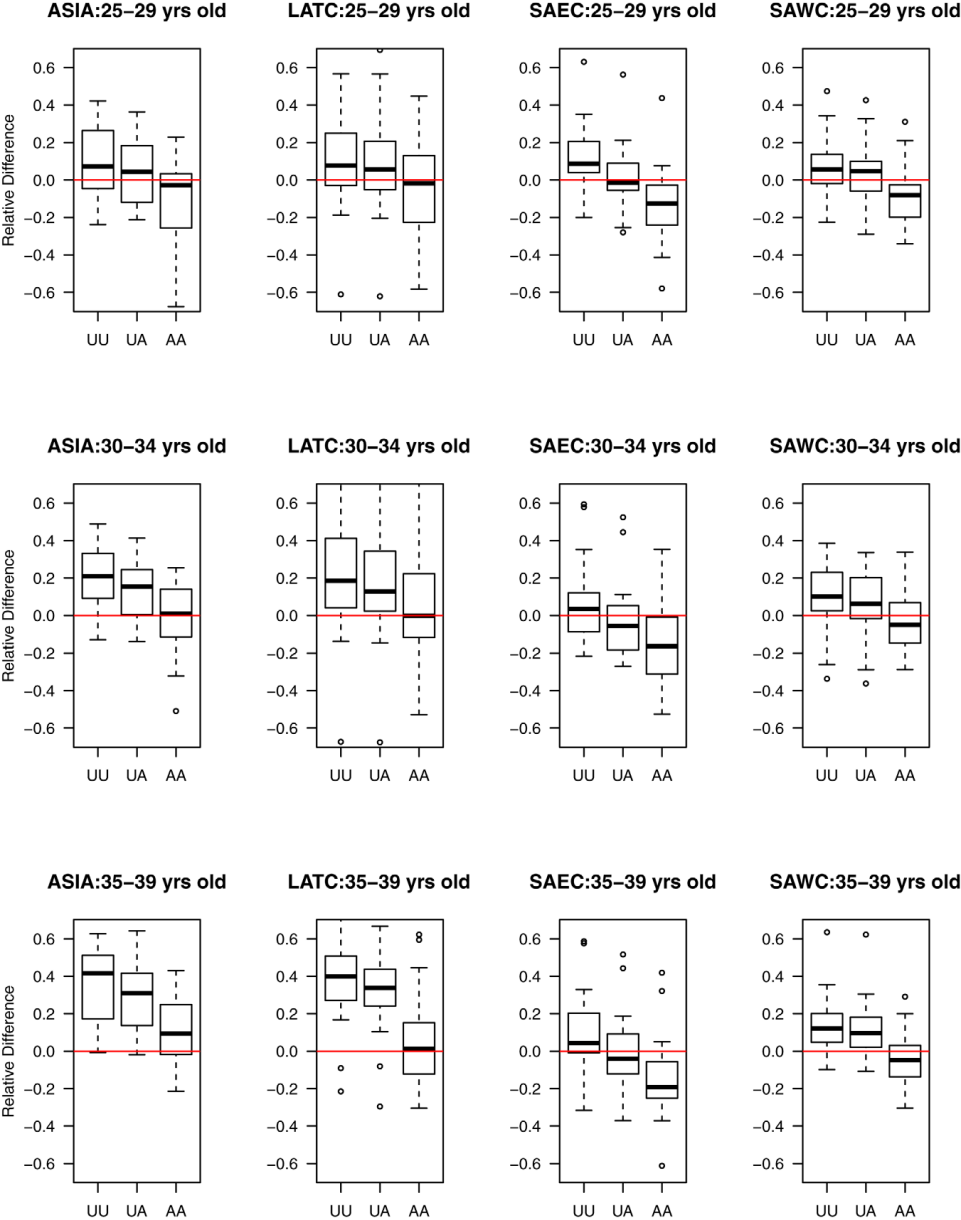
Relative paired differences between under-five mortality rate estimates derived from DHS surveys by age group of mother and geographic region. Regions include South/Southeast Asia (ASIA), Latin America and the Caribbean (LATC), East and Central Africa (SAEC), and West Africa (SAWC). In each panel, the left box plot displays the relative paired difference between raw direct estimates and classical Brass indirect estimates (UU); the middle box plot displays the relative paired difference between direct estimates that have been adjusted for birth transference and classical Brass indirect estimates (UA); and the right box plot displays the relative paired difference between direct estimates after adjustment for birth transference and cohort-parity-based Brass estimates (AA). Source: DHS 1985–2009.

Figure 3 displays the results of a series of successive paired difference tests between direct and indirect estimates calculated from 132 DHS surveys in 49 countries for the maternal age groups 25–29, 30–34, and 25–39 y. The DHS survey data sources used in this analysis are listed in Table 1. Across the three age groups (25–29, 30–34, and 35–39 y) and the four geographic regions, successive adjustment for birth transference and violations of the constant fertility assumption of the Trussell version of the Brass method resulted in increased consistency between direct and indirect estimates, as shown in Figure 3. This result is consistent with the hypothesis that most of the difference between direct and indirect estimates derived from the same DHS birth histories is explained by birth transference and violations of the constant fertility assumption of the Brass indirect method. For Asia, Latin America, and West Africa, adjustment of both direct and indirect estimates (to control for the effects of birth transference and relax the assumption of constant fertility in the recent past, respectively) results in notably more consistent estimates. However, this is not the case for estimates associated with the East African region. For this region, adjustment only of one set of estimates improves consistency. But adjustment of both direct and indirect methods results in estimates that are less consistent than when neither estimate is adjusted. Further, for this region, in contrast to others, adjusted indirect estimates tend to be approximately 10%–20% lower than adjusted direct estimates. Recent literature has noted that identifying fertility trends in several sub-Saharan African countries, particularly East African countries, is challenging and that there are several inconsistent findings amongst recent studies that all rely on DHS data [19]. Depending on the fertility estimation method used, some researchers find a fertility stall where others identify a fertility decline [19]–[25]. Machiyama [19] concluded that these inconsistencies are a function of DHS data being vulnerable to age displacement of children, omission, and other survey errors. Thus, for countries where recent fertility decline is small or nonexistent, the cohort-based parity adjustments may over-adjust the indirect estimates.

#### Detailed examination of consistency between direct and indirect estimates across different health transition typologies

**Figure 4:**
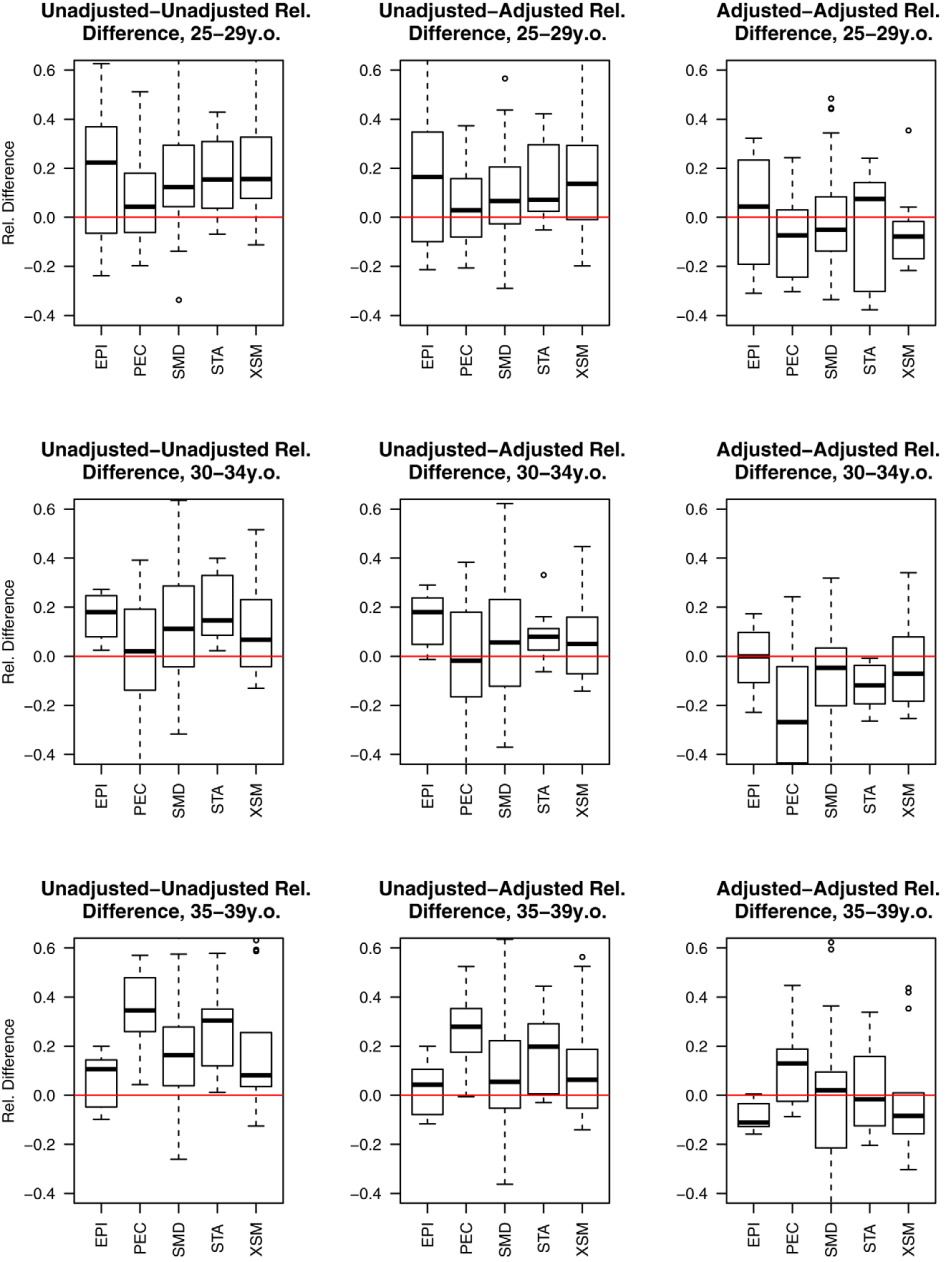
Estimated relative paired differences for under-five mortality rates for developing countries organized by Garenne and Gakusi [**14**] categories. Categories: epidemiologic crisis (EPI), political and economic crisis (PEC), smooth mortality decline (SMD), periods of stagnation (STA), and periods of excess mortality (XSM).

By decomposing health and mortality transitions into different typologies (epidemiologic crisis, political and economic crisis, smooth mortality decline, periods of stagnation, and periods of excess mortality), Garenne and Gakusi were able to identify the key underlying factors that drive variation in health transitions in sub-Saharan Africa [14]. I use their typological framework and extend it to countries in South/Southeast Asia and Latin America. Given the Brass indirect estimation method's assumption of constant fertility in the recent past, this framework is useful to assess the potential impact of violations of the constant mortality assumption of the Brass method on consistency of direct and indirect estimates (after controlling for birth transference and fertility change, respectively). Figure 4 displays the paired relative differences between direct and indirect estimates for countries organized by these typological categories. For countries that experienced a steady mortality decline, the paired relative differences between the direct and indirect estimates associated with birth histories from women aged 25–29, 30–34, and 35–39 y are relatively large, when raw direct and indirect estimates are considered. However, these differences are substantially reduced (approximating zero for birth histories from women aged 25–29 and 30–34 y) after adjusting for birth transference in direct estimates and violations of the Brass method assumptions in indirect estimates. This suggests that for populations that have experienced a steady mortality decline, there is very little difference in using direct and indirect estimates derived from birth histories from women aged 25–29, 30–34, and 35–39 y once appropriate adjustments are made for data errors and violation of the Brass method assumptions. In contrast, for countries that experienced political and economic crises, periods of excess mortality, and periods of stagnation, notable differences remain between direct and indirect estimates even after controlling for data errors in the direct estimates and assumption violations in the Brass indirect method. This result suggests the need to explore these cases in more detail, as presented in the following paragraphs.

**Figure 5:**
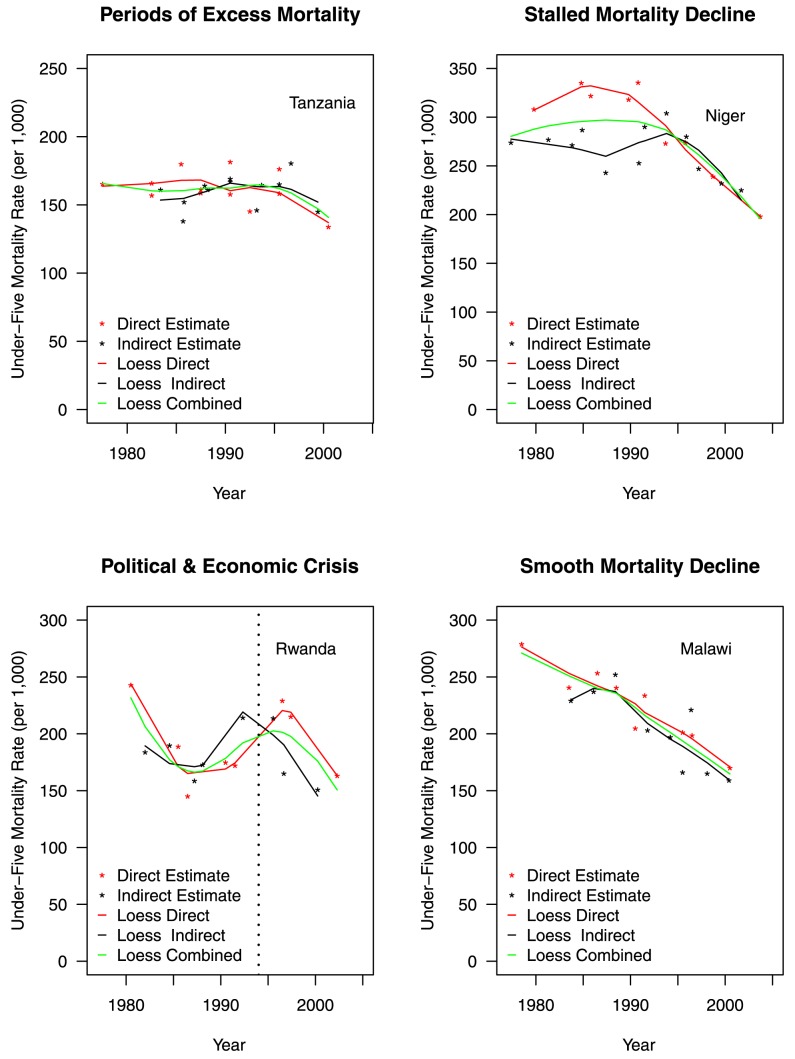
Differences in estimated trends of adjusted under-five mortality rates for example countries organized by Garenne and Gakusi [**14**] categories.

As the Trussell version of the Brass indirect method assumes linear mortality change in recent times, the violation of this assumption is one potential source of inconsistency between direct and indirect estimates. Figure 5 presents example cases showing the consistency of the two types of estimates across different health transition characterizations, after adjustments are made for birth transference (in direct estimates) and fertility change by using cohort-specific parity ratios (in indirect estimates). Point estimates derived using both direct and indirect estimation methods are shown in each graph. In addition, three loess curves are fitted for each country: one through the direct estimates only, one through the indirect estimates only, and one through the full combination of direct and indirect estimates.

For countries that experienced either smooth mortality declines or short periods of excess mortality, such as Malawi and Tanzania in Figure 5, there is little observable difference between general fitted trends using only direct estimates, only indirect estimates, or a combination of the two. However, in the case of populations that have experienced longer periods of excess mortality, the indirect estimates do a poor job of capturing short-term patterns (as seen for Tanzania in the early 1980s). For Niger, a population that experienced a stall in mortality decline through the 1970s, 1980s, and early 1990s, direct estimates capture a general trend of stagnation followed by decline. On the other hand, the indirect estimates are more variable during the period of stagnation, suggesting more variation than their direct counterparts in 1980s and 1990s, and that the onset of mortality decline began only in the mid-1990s. However, after the onset of mortality decline in Niger, both the direct and indirect estimates are generally consistent.

For Rwanda, a country that experienced a political/economic crisis in the 1994 genocide, the adjusted direct and indirect estimates have notable inconsistencies both before and after the elevated mortality period around 1994. The indirect estimates tend to smooth out rapid change in mortality, and therefore underestimate the effect of the 1994 genocide. Further, the mortality peak is dislocated in time (to before the actual genocide), suggesting that in such crisis situations indirect estimates are particularly vulnerable to time-location errors and bias. Further, as Pedersen and Liu [26] note, in another paper in the 2012 *PLOS Medicine* Collection “Child Mortality Estimation Methods,” when examining acute mortality crises such as the Rwandan genocide, under-five mortality rates using 5-y periods also tend to smooth out the genocide-related mortality, unlike when 1-y periods are used [26].

## Discussion

I examined the consistency of direct and indirect methods used to estimate under-five mortality rates when high-quality vital registration data are not available. Assessments were made of direct and indirect estimates derived from the same FBHs (such as those collected in a DHS survey). Specific examination of the relative effects of data errors in FBHs (principally birth transference) and violations of simplifying assumptions in the SBH methods (principally assumptions about fertility and mortality patterns in the recent past) was undertaken.

The main finding from this research is that indirect estimates are generally consistent with direct estimates, once adjustment is made for fertility change and birth transference, but don't appear to add much additional insight beyond direct estimates. However, choice of method does matter—both in the indirect method used and in adjusting for data errors and the constant fertility assumption of the Brass indirect approach. This study strengthens the existing literature on comparisons of direct and indirect estimation of under-five mortality, by drawing on a larger body of empirical data than previous studies and by also successively adjusting estimates for identifiable errors and biases.

When examining relative differences across different health and mortality transitions, greater inconsistencies between direct and indirect estimates were observed for countries that had experienced either a political or economic crisis or a stalled health transition. In contrast, in countries where either smooth mortality declines or only short periods of excess mortality were experienced, the adjusted estimates were generally consistent. These findings suggest the importance of adjustment for birth transference and use of cohort parity data when constructing direct and indirect estimates from FBHs. They also suggest the general consistency of the Brass indirect method during relatively smooth health transitions, but point to its limitations when applied to crisis mortality situations or populations that have experienced stalled health transitions.

The analysis presented here suggests that, when FBHs are available, adjusted indirect estimates are generally consistent with the comparable direct estimates. The basis for adjustment to such indirect estimates is the use of cohort-specific parities from the FBHs, as opposed to the period parities used in the classical Brass method. The clearest inconsistencies between the two estimates are observed in populations whose recent health transitions have departed substantially from a smooth mortality decline. These findings suggest that such indirect methods can be useful but are unlikely to provide deeper insights and more reliable estimates than direct methods, when FBHs are available. This finding lends support to, when FBHs are available, reliance on direct estimates from the FBHs when trying to infer the overall pattern of under-five mortality change over time. However, in the special case when birth transference in FBHs is considerable and both mortality and fertility in the recent past have been approximately constant, indirect estimates may be preferable.

This paper has examined the consistency of direct and indirect estimates from widely available survey data. Retrospective FBHs and SBHs are collected in situations where comprehensive vital registration data are unavailable. As a result, this analysis, like previous empirical investigations into direct and indirect estimates of under-five mortality, is limited by the lack of ground truth data against which SBH and FBH methods could be validated. As a result, this study has focused on consistency between different estimates, as opposed to the validity of the underlying estimates themselves. That being said, there are two natural extensions to this study that are likely to lead to new insights into the remaining questions about the validity and reliability of direct and indirect estimates collected through ad hoc surveys and population censuses.

First, the demographic surveillance sites coordinated by the INDEPTH Network collect high-quality vital registration information in a diverse set of developing country sub-national populations. By carrying out a series of retrospective mortality surveys, in which a SBH is included, researchers can study both the reliability and validity of the SBH methods. Essentially, the fertility and mortality registration data from the demographic surveillance sites can be treated as “ground truth” data. Such a series of studies is likely to lead to new insights into the real-world performance of FBH and SBH methods in different demographic and epidemiological settings.

Second, demographic microsimulation could be used to more explicitly explore some of the main assumptions of the indirect estimation methods. Specifically, through such a simulation testbed framework, the types and magnitudes of error and bias associated with permutations and combinations of data errors and model assumption violations could be examined. Such an examination would be useful to better understand the variability due to model misspecification and data errors associated with estimates derived from SBHs.

## Supporting Information

Text S1 **Decomposition of Brass estimator into model-based error and data error components.**
(PDF)

## Abbreviations

DHS: Demographic and Health Surveys
FBH: full birth history
SBH: summary birth history
