# Supplementary material for: Child Mortality Estimation: Consistency of Under-Five Mortality Rate Estimates Using Full Birth Histories and Summary Birth Histories
Source: PLoS Med. 2012 Aug 28;9(8):e1001296. doi: 10.1371/journal.pmed.1001296 (PMC3429405; doi:10.1371/journal.pmed.1001296)
Supplement: Text S1 — Decomposition of Brass estimator into model-based error and data error components. (PDF) [file pmed.1001296.s001.pdf]

Text S1: Decomposition of Brass Indirect Estimator into Component Parts

The Brass indirect estimation method is used to estimate the probability of dying before a given age, M, denoted as $q(M)$ in the following way:

$$\hat{q}(M) = k.D_x \tag{1}$$

where D_x is the proportion of dead children born to mothers of a given age, x, and k is an estimated constant used to transform the observed data on D_x to $q(M)$.

The proportion of deceased children can be written in the following way:

$$D_x = \int_0^{x-\alpha} c_x(a)q(a)da \tag{2}$$

where $c_x(a)$ is the relative frequency of children at age a for women aged x and $q(a)$ is the probability of dying between birth and age a in the reference population. In reality, as we cannot measure D_x exactly, we estimate it using survey data (like those collected in a DHS) as follows:

$$D_x \approx \int c_{x,s}(a)q_s(a)da \tag{3}$$

where $c_{x,s}(a)$ is the relative frequency of children at age a in the survey population and $q_s(a)$ is the probability of dying between birth and age a in the survey population.

The conversion constant, k, used to transform the proportion of deceased children into the mortality statistics $q(x)$ can be written as:

$$k = \frac{q_M(M)}{\int c_{x,M}(a)q_M(a)da} \tag{4}$$

where $c_{x,s}(a)$ is the relative frequency of children at age a and $q_s(a)$ is the probability of dying between birth and age a as per a particular set of fertility and mortality model specifications.

By combining equations **??** and **??**, we can rewrite the Brass indirect estimation equation as follows:

$$\hat{q}(M) \approx \overbrace{\frac{q_M(M)}{\int c_{x,M}(a)q_M(a)da}}^{Model-Based} \underbrace{\int c_{x,s}(a)q_s(a)da}_{Survey-Based} \tag{5}$$

This representation of the Brass indirect estimation method, shown in Equation **??**, helps us to explicitly identify which parts of the estimator is subject to model uncertainty and which parts of the estimator are vulnerable to data errors and biases. Specifically, that model-based error and bias is generated from the component $D_x \approx \int c_{x,s}(a)q_s(a)da$ and survey-based error results from the component $k = \frac{q_M(M)}{\int c_{x,M}(a)q_M(a)da}$.

If the model parameters and data are error-free, then the estimate is correct such that

$$\hat{q}(M) = q(M) \tag{6}$$

$$= \frac{q(M)}{\int c_x(a)q(a)da} D_x \tag{7}$$
